# Light-Mediated 3D-Printed Wound Dressings Based on Natural Polymers with Improved Adhesion and Antioxidant Properties

**DOI:** 10.3390/polym17081114

**Published:** 2025-04-20

**Authors:** Rute Silva, Matilde Medeiros, Carlos T. B. Paula, Sofia Saraiva, Rafael C. Rebelo, Patrícia Pereira, Jorge F. J. Coelho, Arménio C. Serra, Ana C. Fonseca

**Affiliations:** 1 CEMMPRE, ARISE, Department of Chemical Engineering, University of Coimbra, Rua Sílvio Lima-Polo II, 3030-790 Coimbra, Portugal; rute.silva@student.uc.pt (R.S.); uc2019237674@student.uc.pt (M.M.); uc44571@uc.pt (S.S.); rafaelr@eq.uc.pt (R.C.R.); papereira@ipn.pt (P.P.); jcoelho@eq.uc.pt (J.F.J.C.); aserra@eq.uc.pt (A.C.S.); 2 IPN, Instituto Pedro Nunes, Associação para a Inovação e Desenvolvimento em Ciência e Tecnologia, Rua Pedro Nunes, 3030-199 Coimbra, Portugal

**Keywords:** 3D printing, digital light processing (DLP), wound healing, hydrogel, micropatterned structures, adhesion properties, antioxidant properties

## Abstract

The lack of personalized wound dressings tailored to individual needs can significantly hinder wound healing. Hydrogels offer a promising solution, as they can be engineered to mimic the extracellular matrix (ECM), providing an optimal environment for wound repair. The integration of digital light processing (DLP), a high-resolution 3D printing process, allows precise customization of hydrogel-based wound dressings. In this study, gelatin methacrylate (GelMA)-based formulations were prepared in combination with three different polymeric precursors: methacrylated hyaluronic acid (HAMA), poly (ethylene glycol) diacrylate (PEGDA) and allyl cellulose (MCCA). These precursors were used to print high-resolution micropatterned patches. The printed constructs revealed a high gel content and a good resistance to hydrolytic degradation. To improve the adhesive and antioxidant properties of the printed patches, gallic acid (GA) was incorporated through surface functionalization. This enabled the scavenging of approximately 80% of free radicals within just 4 h. The adhesive properties of the printed wound dressings were also significantly improved, with further enhancement observed upon the addition of Fe^3+^ ions. In vitro cytocompatibility tests using a fibroblast (NHDF) cell line confirmed the suitability of the materials for biomedical applications. Thus, this study demonstrates the potential of DLP-printed hydrogels as advanced personalized wound dressing materials.

## 1. Introduction

Skin, recognized as the largest organ in the human body, plays a vital role in maintaining homeostasis and provides a protective barrier against external agents. When the skin is injured, an inflammatory response is initiated at the wound site to restore its essential barrier function [1]. The wound healing process is inherently complex and involves a series of molecular and cellular events that can lead to either tissue regeneration or repair [2]. In healthy individuals, wound closure is a natural process; however, various conditions, including chronic illnesses, infections and severe injuries, can hinder healing, leading to chronic wounds. This problem affects around 40–60 million people worldwide, representing a cost of around USD 28 billion to the healthcare systems. Moreover, the biomaterials market for wound treatment is growing rapidly and is expected to reach USD 30.5 billion by 2030 [3]. These values highlight the importance of developing advanced wound dressing materials to facilitate and accelerate recovery [4]. Although traditional wound dressings, such as gauze and cotton wool, provide an effective physical barrier against pathogen entry and can absorb wound exudate, they often fail in creating optimal healing conditions, particularly in the case of chronic wounds, which frequently demand more sophisticated and personalized therapeutic strategies [5].

The limitations of traditional wound dressings have driven the search for innovative solutions, including the adoption of 3D printing technologies. These advanced manufacturing techniques allow for the precise customization of wound dressings, tailoring their properties to the specific requirements of individual patients and diverse wound types [6,7,8]. They also enable the fabrication of materials that mimic the extracellular matrix (ECM) properties (e.g., mechanical, porosity, topology), thereby providing an ideal microenvironment for cell migration, proliferation and tissue regeneration [9]. Digital light processing (DLP), a type of 3D printing process, offers the possibility of designing micropatterned structures that are known to improve tissue adhesion by increasing the surface area and establishing direct contact with damaged tissues [10]. These possibilities, combined with the possibility of incorporating bioactive molecules, such as drugs and growth factors, during the 3D printing make DLP-based systems a powerful approach to significantly enhance the healing process [11].

Among the materials used for the development of these advanced dressings, methacrylated gelatin (GelMA) has attracted significant attention because of its ECM-mimicking properties, excellent biocompatibility and tunability [12]. Despite these advantages, GelMA alone can exhibit printability issues and limited mechanical properties, requiring its combination with other polymers to improve its performance [13]. Camci-Unal et al. demonstrated that the incorporation of methacrylated hyaluronic acid (HAMA) into GelMA resulted in a significant improvement in the mechanical properties compared to both individual hydrogels [14]. The same observation was made by Wang et al., who showed that the addition of poly(ethylene glycol) diacrylate (PEGDA) to GelMA did in fact provide improved mechanical properties without compromising cell viability [15].

In this work, GelMA-based formulations were prepared in combination with three different polymeric precursors, namely HAMA, PEGDA and allyl cellulose (MCCA), and subjected to 3D printing via DLP. Cellulose, despite not being a constituent of the human body, has received a lot of attention, as it is the most abundant natural polymer on earth and is reported to be biocompatible [16,17]. Moreover, cellulose and its derivatives present outstanding properties for wound management, including efficient absorption and retention of wound exudates, which contribute to maintaining an optimal moisture environment essential for the effective regeneration of chronic wounds [18,19,20]. It is worth mentioning that, to the best of our knowledge, this is the first time that a photopolymerizable cellulose derivative is combined with GelMA for the development of formulations for DLP printing toward wound dressing.

Additionally, the 3D-printed constructs were functionalized with gallic acid (GA) to enhance their structural integrity and therapeutic potential, further expanding the functional versatility of the patches [21]. Gallic acid (GA) is a natural polyphenol widely found in nature. This polyphenolic compound has been shown to have strong pharmacological activities, such as anti-inflammatory, antimicrobial, antitumor and, more importantly, antioxidant effects. When incorporated into hydrogels, it can improve their mechanical strength and adhesiveness. As a result, GA helps to create a secure interface between the dressing and the wound, protecting tissues from further damage [21,22,23].

This study explored the development of high-resolution micropatterned patches using 3D printing processes designed to improve adhesion to irregular and deep wound beds. Four formulations were prepared by combining GelMA with HAMA, PEGDA or MCCA to obtain 3D constructs with tailored properties. The integration of different polymers into the formulation allowed the optimization of the printability, mechanical integrity and biocompatibility of the materials. The incorporation of GA allowed the improvement of the antioxidant capacity and adhesiveness. These advanced dressings demonstrated significant potential to support tissue regeneration based on in vitro assessments.

This study highlights the potential of combining light-based 3D printing processes with ECM-derived natural polymers to create personalized wound dressings with enhanced biocompatibility, adhesion and efficacy.

## 2. Materials and Methods

### 2.1. Materials

Gelatin type A (gel strength ≈ 300 g Bloom), microcrystalline cellulose (MCC) (Avicel PH-101, particle size ≈ 50 μm), methacrylic anhydride (94.0%) and phosphate-buffered saline (PBS) tablets were obtained from Sigma-Aldrich (St. Louis, MO, USA). Poly(ethylene glycol) diacrylate (PEGDA, *n* ≈ 14), gallic acid monohydrate (GA) (>98.0%), allyl glycidyl ether (AGE) (>99.0%), lithium phenyl(2,4,6-trimethylbenzoyl)phosphinate (LAP) (>98.0%), acid yellow 23 (Tartrazine) (>98.0%), N-hydroxysuccinimide (NHS) (>98.0%), 2,2-diphenyl-1-picrylhydrazyl (DPPH) (>97%) and 2-morpholinoethanesulfonic acid (MES) (>99.0%) were all acquired from TCI Chemicals (Zwijndrecht, Belgium). Hyaluronic acid sodium salt (HA) (MW ≈ 40–50 kDa) and 1-(3-dimethylaminopropyl)-3-ethylcarbodiimide (EDC) were purchased from Biosynth (Staad, Switzerland). Urea (99.5–100.5%) was acquired from ChemLab (Zedelgem, Belgium). Sodium hydroxide (NaOH), ethanol (96%) and acetone (>99%) were obtained from José Manuel Gomes dos Santos (Odivelas, Portugal). Deuterium oxide (99.90%) was purchased from EurIsotop (Saint-Aubin, France). All the reagents were used as received. Deionized water was obtained by reverse osmosis.

### 2.2. Methods

#### 2.2.1. Functionalization of the Natural Polymers

##### Functionalization of Gelatin with Methacrylic Anhydride (GelMA)

**Scheme 1:**
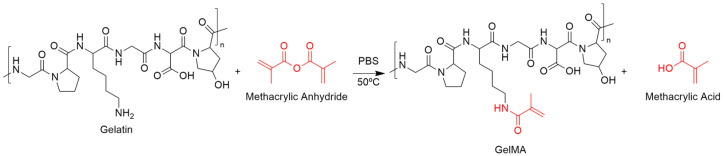
Schematic illustration of the functionalization of gelatin with methacrylic anhydride (MA).

The functionalization of gelatin with methacrylic anhydride (GelMA) was performed following previously reported protocols (Scheme 1) [24]. In a 100 mL round-bottom flask, immersed in a bath at 50 °C, 5 g of type A gelatin was dissolved in 50 mL of 10 mM phosphate-buffered saline (PBS) solution (pH 7.4) to obtain a 10 wt. % gelatin solution. After dissolving the gelatin, 1 mL (7 mmol) (~3.5 eq.) of methacrylic anhydride (MA) was slowly added to the solution. The reaction was kept at 50 °C, under magnetic stirring, for 3 h. After this time, the reaction product was dialyzed (dialysis membrane MWCO = 12–14 kDa) against distilled water at 40 °C for 7 days and then freeze-dried. The reaction yield was around 80%.

##### Functionalization of Hyaluronic Acid with Methacrylic Anhydride (HAMA)

**Scheme 2:**
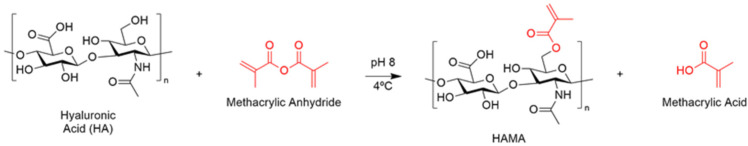
Schematic illustration of the functionalization of hyaluronic acid with methacrylic anhydride (MA).

HAMA was synthesized as previously described [25]. Briefly, 1 g of HA (low molecular weight, 40–50 kDa) was dissolved in 100 mL of deionized water in a 250 mL round-bottom flask. The pH of the solution was adjusted to 8 using a 1 M NaOH solution. Subsequently, 4 mL (27 mmol) (10 eq.) of MA was added to the solution using a feeding pump at a rate of 10 mL/h in an ice bath. Once MA was added, the pH of the solution was maintained at 8 using a 1 M NaOH solution (Scheme 2). The reaction was carried out for 24 h at room temperature. Then, 100 mL of 0.5 M NaCl was added to the solution, which was then precipitated in cold ethanol and filtered. The filtered product was dissolved and dialyzed against distilled water (dialysis membrane MWCO = 12–14 kDa) for 7 days, and the final product was obtained by freeze-drying. A reaction yield of around 85% was obtained. 

##### Functionalization of Microcrystalline Cellulose with Allyl Glycidyl Ether (MCCA)

**Scheme 3:**
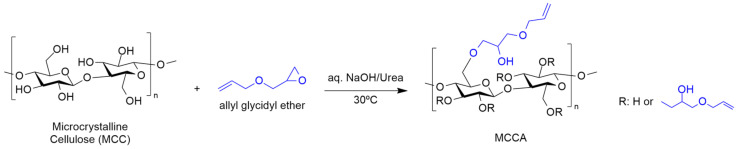
Schematic illustration of MCC functionalization with allyl glycidyl ether (AGE).

Microcrystalline cellulose (MCC) was functionalized according to our previously reported protocol [26] (Scheme 3). First, a 6 wt. % cellulose transparent solution was prepared. In order to achieve this, in a 250 mL beaker, 6 g of MCC was slowly added to 100 mL of a cold-prepared solvent solution (~5 °C) containing 7 wt% NaOH and 12 wt% urea and stirred for 15 min. The resulting suspension was placed overnight in a freezer. Subsequently, the beaker was left at room temperature under stirring (400 rpm) until thawing, yielding the MCC solution [27]. Then, the solution was placed in a 250 mL double-necked round-bottom flask and placed in a water bath at 30 °C. Then, 21.95 mL (0.186 mol) of allyl glycidyl ether (AGE) (5 eq./anhydroglucose unit (AGU)) was added dropwise, and the modification reaction was allowed to proceed for 24 h under nitrogen atmosphere. The reaction product was washed with cold acetone to remove excess AGE; the acetone was poured off, and the product was redissolved in 100 mL of deionized water. The reaction product was dialyzed (dialysis membrane MWCO = 3.5 kDa) against deionized water at room temperature until a neutral pH was achieved (~5 days) and then freeze-dried.

#### 2.2.2. Characterization of the Polymeric Precursors

##### ^1^H NMR Spectroscopy

^1^H NMR spectra were obtained using a Malvern Bruker Avance III 400 MHz spectrometer equipped with a 5 mm triple detention probe. Samples of GelMA, HAMA and MCCA were dissolved in deuterated oxide (D_2_O) at a concentration of 15 mg.mL^−1^. The spectra were acquired at 25 °C for HAMA and MCCA and at 30 °C for GelMA.

The degree of substitution of each functionalized polymer was calculated using the respective equations, as presented in Supporting Information (Figures S1–S3).

##### ATR-FTIR Spectroscopy

ATR-FTIR spectra were obtained using an Agilent Technologies Carey 630 spectrometer with a Golden Gate Single Reflection Diamond ATR. The samples were carried out in a range of 4000–750 cm^−1^ with a spectral resolution of 4 cm^−1^ and 64 accumulations.

#### 2.2.3. Preparation and Characterization of the DLP Formulations

The DLP formulations were prepared by dissolving the functionalized polymers, at different ratios, in 10 mM PBS solution (pH 7.4). After optimization, the final polymer concentration was set at 12.5% (*w*/*v*). Stock solutions of LAP (photoinitiator, PI) and tartrazine (photoabsorber, PA) were prepared at concentrations of 100 mM and 10 mM, respectively, and added to the DLP formulations to achieve the desired concentrations of 0.50 wt% for the PI and 0.05 wt% for the PA. The resulting formulations were stirred at 50 °C until a homogeneous solution was obtained. A solution of 12.5% (*w*/*v*) GelMA, with the same amount of PI and PA, was used as the control formulation. Table 1 provides the composition of the studied formulations in terms of quantities of the precursors, PI and PA.

##### Photorheology

Rheological measurements were performed using a Kinexus Prime Pro + rheometer (Malvern) equipped with an OmniCure S2000 UV Curing System (320–500 nm). The photorheological tests were performed in parallel-plate geometry (20 mm diameter; 0.1 mm gap). Measurements were conducted at 40 °C. Oscillatory single-frequency (1 Hz) measurements were performed to determine the viscoelastic properties associated with the crosslinking kinetics. After 30 s of system stabilization, the UV light was turned on at an intensity of 125 mW cm^−1^ for 10 s, irradiating the DLP formulation from the bottom through a quartz glass plate. The evolution of the elastic modulus (G’) during irradiation was recorded to determine the crossover point.

#### 2.2.4. 3D Printing Process

All the hydrogel-printed samples were obtained using a Lumen X^TM^ printer (Cellink, Gothenburg, Sweden). This light-based printer is equipped with a blue light source (405 nm, LED) that operates within an intensity range of 10–30 mW/cm^2^. The printer offers a pixel resolution of 50 μm in the xOy plane and a resolution of 50 μm in the z-plane.

##### 3D Models

**Figure 1:**
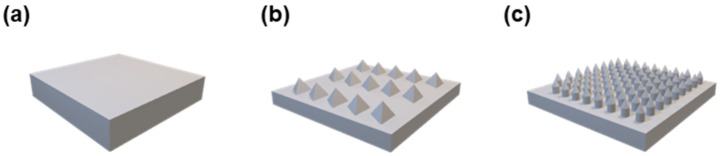
Representation of the 3D CAD models: Smooth surface (**a**), Micro-pyramids (**b**) and Micro-spikes (**c**).

The desired 3D structures were designed using a CAD software (Fusion 360 v. 2.0.21286). Three different types of 1 × 1 cm^2^ patch models were designed with distinct microstructures on the top surface: a patch with a smooth surface, a patch with micro-pyramids and a patch with micro-spikes (Figure S1). The height of the microstructures was set to 0.2 cm along the z-axis. A representation of the 3D CAD models is shown in Figure 1. The CAD models were then transformed into an STL file and exported to Lumen X^TM^, where inbuilt software was employed to slice the 3D models into 100 μm thick layers.

##### Printing Parameters

The printing parameters were set to a light power intensity of 16.5 mW/cm^2^, 15 s of exposure time, with a burn-in exposure time of 60 s for the first two layers. The bed temperature of the vat was set and maintained at 45 °C to prevent the gelation of the resin during the printing process.

#### 2.2.5. Characterization of the Printed Constructs

##### Gel Content

The printed constructs were dried directly after printing in an oven to remove all the water from the network. The dry weight of each sample was determined by using a high-precision balance. The printed constructs were then immersed in 10 mM PBS solution (pH 7.4) and incubated at 37 °C. The PBS solution was changed at least four times over 24 h to ensure that the patches were properly washed. The washed hydrogel patches were then dried in an oven to obtain their dry weights.

The percentage gel content of the hydrogels was calculated using Equation (1):

(1) $$Gel\;content\;(\%)=\frac{W_{t}}{W_{0}}\times 100$$

where *W_t_* corresponds to the dry weight of the samples after washing in PBS solution, and *W*_0_ is the initial dry weight of the samples before immersion in PBS solution [28].

##### Hydrolytic Degradation

Degradation studies were performed on all the hydrogel formulations. The hydrogel samples were dried in a vacuum oven at 50 °C to remove all water from the network. The dry weight of each sample was determined using a high-precision balance. To study the effect of degradation on each hydrogel formulation, the samples were immersed in 10 mM PBS solution (pH 7.4) at 37 °C and withdrawn at different time points [29]. The time points were set to 1, 3, 7 and 14 days. After withdrawing the hydrogel from the solution, the samples were dried in a vacuum oven at 50 °C until a constant weight was obtained.

The degradation of the hydrogels was determined by calculating the percentage weight loss of the hydrogel using Equation (2).

(2) $$Weight\;loss\;\left(\%\right)=\frac{W_{0}-W_{t}\;}{W_{o}}\times 100$$

where *W*_0_ corresponds to the initial dry weight of the samples before immersion in PBS solution, and *W_t_* corresponds to the weight of the sample measured after the determined time interval (*t*). The final values were obtained using three replicates of each sample.

##### Surface Morphology

The surface morphology of the 3D-printed hydrogel constructs was examined using three different printed models to verify the resolution of the DLP printer using the prepared formulations. The samples were dried in a vacuum oven at 50 °C for 48 h, and then, the surface was sputter-coated with a conductive gold layer. The samples were analyzed at different magnifications using a field emission scanning electron microscope (FE-SEM), ZEISS MERLIN Compact/VPCompact, Gemini II, with 2.00 kV acceleration voltage.

#### 2.2.6. Functionalization of the Printed Constructs with Gallic Acid (GA)

**Scheme 4:**
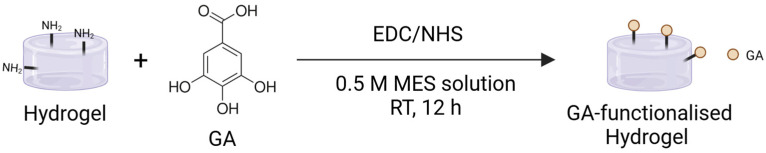
Schematic illustration of the hydrogel functionalization with GA.

First, the hydrogel constructs were washed in a 10 mM PBS solution (pH 7.4) and dried in an oven. Next, in a beaker protected from light, 0.47 g (3 mmol) of EDC was dissolved in 10 mL of a 0.5 M MES solution (pH 5). Once dissolved, 0.35 g (3 mmol) of NHS was added to the solution. After dissolution, 0.17 g (1 mmol) of gallic acid (GA) was added to the activating agents solution and left under stirring for 1 h. After this time, the dried patches were immersed in the 0.1 M GA solution and left under low stirring overnight. Finally, the hydrogels were washed with a 0.5 M MES solution (pH 5) and again with a 10 mM PBS solution (pH 7.4) (Scheme 4).

#### 2.2.7. Antioxidant Capacity of the Printed Constructs

The antioxidant properties of the printed constructs functionalized with GA were evaluated for all formulations. To evaluate the scavenging response of ROS species, the hydrogel patches modified with GA were immersed in a 25 μM DPPH ethanol solution. Subsequently, at different time points, the absorbance spectrum of the DPPH solution was obtained using UV–VIS spectroscopy, measured at 517 nm and 10 mm^2^ quartz cuvette cells, using ethanol as a reference [30].

The antioxidant properties of the printed constructs were evaluated using Equation (3).

(3) $$DPPH\;scavenging\;activity\;(\%)=\frac{\left(A_{control}-A_{sample}\right)}{A_{control}}\times 100$$

where *A_control_* corresponds to the absorbance of the original DPPH ethanol solution, and *A_sample_* corresponds to the absorbance of the solution of the immersed hydrogel sample.

#### 2.2.8. Adhesion Capacity of the Printed Constructs

Adhesion tests were divided into two complementary experiments. The first experiment focused on the influence of the printed construct’s surface morphology. For each resin formulation, three different construct models were printed: a smooth-surface construct, a micro-pyramid construct and a micro-spike construct. The second experiment was performed to evaluate the influence of the adhesion of constructs modified with GA. In addition, the influence of the adhesion provided by the application of a 100 mM FeCl_3_ solution between the patch and the contact surface was also evaluated. In both experiments, the adhesion capacity was tested on different contact surfaces, namely a glass surface, a metal surface, paper and porcine skin.

The adhesive properties of the GelMA and GelMA:MCCA hydrogels were determined using a Kinexus Prime Pro + rheometer (Malvern) by performing a pull-away test using rSpace for Kinexus software version 1.76 [31]. In this test, tackiness and adhesion were assessed by measuring the normal force (N) over time (s) as the upper plate was retracted from the hydrogel patches. The test was conducted on cylindrical hydrogel samples with a diameter of 20 mm and a thickness of 2 mm. A parallel-plate geometry (20 mm diameter) was used. The vertical gapping parameters were set as follows: an initial gap of 1 mm, a gapping speed of 1 mm/s and a final gap of 30 mm. All measurements were performed at 25 °C, with three replicates measured for each sample.

#### 2.2.9. In Vitro Cytocompatibility of 3D-Printed Hydrogels

##### Indirect Method

The possible cytotoxicity of the leached products of the printed constructs was determined by an indirect method based on ISO 10993-5:2009-Biological evaluation of medical devices [32]. To obtain the degradation products, the sterilized printed structures (via UV light irradiation for 45 min) were immersed in culture medium and maintained at 37 °C for 1, 3, 7, 10, 14 and 21 days. Normal Human Dermal Fibroblasts (NHDF, Sigma-Aldrich) were selected as a cell model and cultured in Minimal Essential Medium (MEM, HyClone™, Cytiva (Marlborough, MA, USA)) supplemented with 10% (*w*/*v*) heat-inactivated fetal bovine serum (FBS, Biowest) and 1% (*w*/*v*) penicillin-streptomycin (SP, Biowest). The cells were maintained at 37 °C in a humidified atmosphere containing 5% CO_2_. To assess the cytotoxicity of the degradation products, NHDF cells (at passages 3–10) were seeded into 96-well cell culture plates at a density of 1 × 10^4^ cells/well in MEM supplemented with 10% FBS and 1% SP. The cells were maintained in an incubator for 24 h at 37 °C and 5% CO_2_, so that the cells adhered to the bottom of the plate and reached confluence. After 24 h, the medium was removed and replaced with 100 μL of the extracts and incubated for 48 h. Cell viability was determined via quantitative analysis using the colorimetric Resazurin-based In Vitro Toxicology Assay Kit (Sigma-Aldrich), according to the manufacturer’s instructions. Briefly, the complete culture medium in each well was replaced with 90 μL of serum-free culture medium and 10 μL of Resazurin reagent, and then, the cells were incubated for a period of 3 h at 37 °C. Subsequently, 100 μL (in triplicate) of the solution from each well was transferred to a 96-well plate, and the absorbance was measured at 570 and 600 nm using a microplate reader. The untreated NHDF cells (i.e., cells seeded directly on the surface of the wells without extracts of the degradation products) served as a negative control. Cell viability was calculated as a percentage relative to untreated NHDF cells, which were considered to have 100% viability.

##### Direct Method

To assess the in vitro effects of the printed hydrogels (after sterilization with UV light irradiation for 45 min) on cell viability and proliferation, NHDF cells at a density of 2 × 10^4^ per well were seeded on the surface of each sample. Then, the cell-seeded hydrogels were maintained at 37 °C and 5% CO_2_ for 3 days. After culturing the cells for a period of 3 days, cell viability in the presence of the hydrogels was also determined using the colorimetric Resazurin-based In Vitro Toxicology Assay Kit (Sigma-Aldrich), according to the manufacturer’s instructions. A positive control for cytotoxicity was performed (K+, cells in a cytotoxic environment, i.e., cells treated with 10% (*w*/*v*) DMSO).

##### Statistical Analysis

All experiments were conducted in triplicate, and data were presented in the form of mean ± standard deviation (SD). Significant differences within each experimental group were determined through one-way analysis of variance (ANOVA), followed by Tukey’s multiple comparisons test. A *p*-value lower than 0.05 (**** *p* < 0.0001) was considered as being statistically significant. Data analysis was performed in GraphPad Prism version 10.4 XML Project (GraphPad Software Inc., La Jolla, CA, USA).

## 3. Results and Discussion

### 3.1. Synthesis and Characterization of the Polymeric Precursors

#### Functionalization of the Polymeric Precursors

Gelatin was functionalized with MA to obtain the photopolymerizable methacrylated gelatin (GelMA) [33]. The functionalization reaction is presented in Scheme 1.

**Figure 2:**
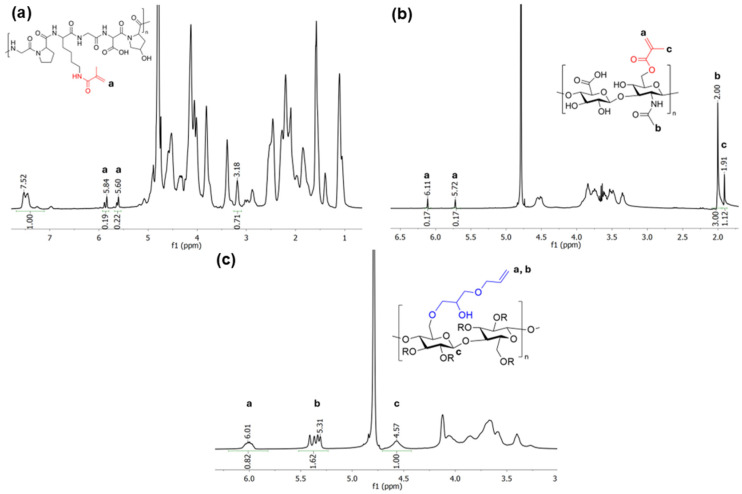
^1^H NMR spectra of GelMA (**a**), HAMA (**b**) and MCCA (**c**) in D_2_O. The lowercase letters identify the protons of interest in the structure of the polymeric precursors.

The chemical structure of the synthesized GelMA was assessed via ^1^H NMR spectroscopy (Figure 2a). The ^1^H NMR spectrum of GelMA exhibits two new peaks at δ 5.52 and δ 5.75 ppm (a), corresponding to the protons of the double bonds of the methacrylate groups. Concomitantly, a decrease in the integral of the peak at δ 3.10 ppm, associated with a decrease in the lysine unit in the gelatin backbone, is observed (Figure S2). This suggests that the reaction of gelatin with MA was successful [34].

HA was functionalized with MA to obtain photopolymerizable methacrylated hyaluronic acid (HAMA). The functionalization reaction is presented in Scheme 2. The chemical structure of the synthesized HAMA was assessed via ^1^H NMR spectroscopy (Figure 2b). The ^1^H NMR spectrum of HAMA exhibits two peaks at δ 5.72 and δ 6.11 ppm (a), corresponding to the protons of the double bonds of the methacrylate groups, indicating that the methacrylate groups were successfully attached to the hydroxyl group of HA [35].

MCC was functionalized with AGE to obtain the photopolymerizable allyl microcrystalline cellulose (MCCA) [36]. The functionalization reaction is presented in Scheme 3. The chemical structure of the functionalized MCCA was obtained via ^1^H NMR spectroscopy (Figure 2c). The ^1^H NMR spectrum of MCCA exhibits two peaks at δ 5.31 and δ 6.01 ppm (a,b), which are assigned to the double bonds of the allyl groups, confirming that the functionalization of MCCA was successful [37].

The degree of functionalization (DS) of the polymeric precursors was determined following the equations presented in the Supporting Information. The values are summarized in Table 2.

The degree of functionalization of GelMA was obtained by comparing the area of the peak of the -CH_2_ protons belonging to the residual lysine moieties in the ^1^H NMR spectrum of GelMA and the area of the peak of the -CH_2_ protons belonging to the lysine moieties in the ^1^H NMR spectrum of native gelatin [25]. To allow comparison between spectra, a normalization was performed on the signal ascribed to the protons of the L-phenylalanine (6.9–7.4 ppm) residues in gelatin and the GelMA structure (Figure S2).

The degree of functionalization of HAMA was obtained by comparing the integral of the peak belonging to the protons of the methyl group of MA (1.91 ppm (c)) and the integral of the peak associated with the protons of the methyl group (2.00 ppm (b)) in the HA backbone (Figure S3).

The degree of functionalization of MCCA was obtained by comparing the integral of the peak ascribed to the anomeric protons of glucose residues in the cellulose backbone (4.57 ppm (c)) with the integral of the peak corresponding to the protons of the double bonds ((a,b)) (Figure S4).

**Figure 3:**
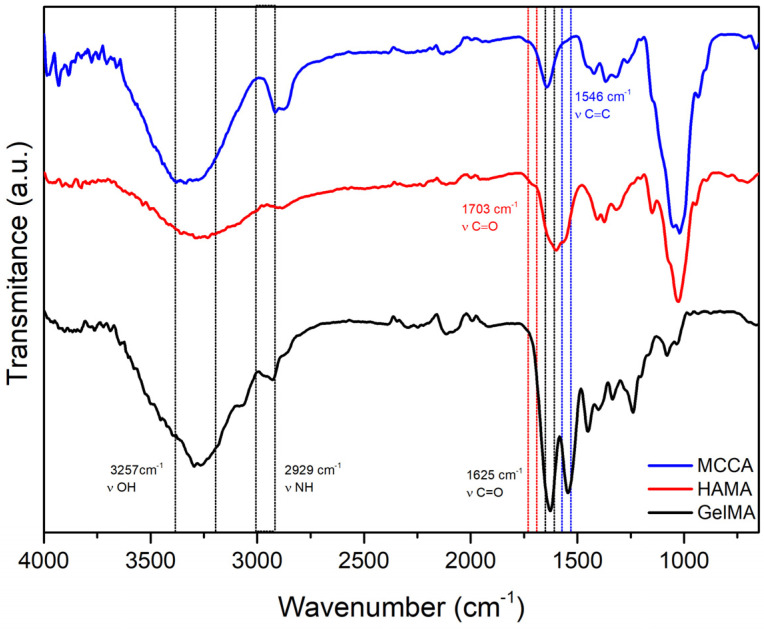
ATR-FTIR spectra of GelMA, HAMA and MCCA.

GelMA, HAMA and MCCA were also characterized by ATR-FTIR spectroscopy to verify their chemical structures, and the spectra are presented in Figure 3.

The FTIR spectrum of GelMA presents the characteristic stretching vibration of the hydroxyl (ν O-H) and amide group (ν N-H) at 3257 cm^−1^ and 2921 cm^−1^, respectively. The absorption band of the stretching vibration of the carbonyl group (ν C=O) belonging to the amide linkage is observed at 1625 cm^−1^. To confirm the functionalization, it is possible to observe the presence of ν C=C bands in the 1550 cm^−1^ region. This confirms the reaction of the methacrylate group with the amine groups in gelatin. In the FTIR spectrum of HAMA, it is possible to observe the band corresponding to the stretching vibration of the carbonyl group (ν C=O) of the ester linkage at 1703 cm^−1^, indicating the successful reaction of the -OH groups of HA with MA.

Regarding the FTIR spectra of MCCA, the main peaks of cellulose backbone are observed, and the peak at ~2900 cm^−1^ (υ C-H) presents a double peak, indicating the structural modifications in C–H bonds [38,39]. It is also possible to observe a small peak at 1546 cm^−1^ associated with the υ C=C, indicating the successful functionalization of cellulose with the allyl groups.

### 3.2. Assessment of the Photoreactivity of the Precursors Toward the Acquisition of the Hydrogels

First, the functionalized polymers were evaluated in terms of their photoreactivity under conditions mimicking those of the DLP 3D printer. To this end, aqueous solutions of the individual precursors (GelMA, HAMA, MCCA and commercial PEGDA), as well as their blends at different ratios, were prepared and subjected to a curing reaction in a curing chamber (Form Cure, Formlabs) operating at 405 nm irradiation. The concentrations of the photoinitiator (PI) and photoabsorber (PA) were set at 0.5 (wt%) and 0.05 (wt.%), respectively. The solutions, with different concentrations, were prepared in PBS (10 mM, pH 7.4). Then, vats with 0.5 mL resin were placed in a pre-heated chamber at 40 °C and left to cure. The gelation of the different formulations was evaluated at different time points using the inverted tube test method.

**Figure 4:**
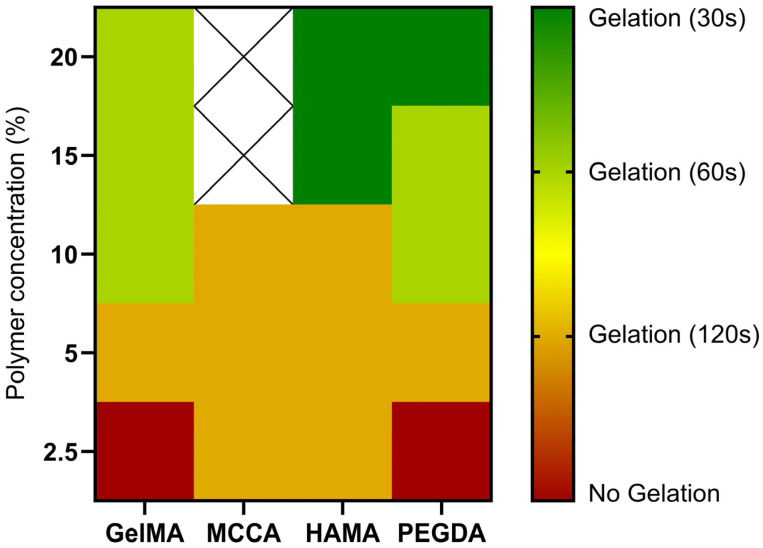
Curing time of the tested formulations under conditions mimicking those of the DLP 3D printer.

Table 3 and Figure 4 provide an overview of the gelation behavior of the different formulations.

These results enabled a preliminary screening of the formulations, revealing that to achieve a crosslinking rate compatible with the time frame required for 3D printing, the main component of the formulation (GelMA) should be set above 10% (*w*/*v*). Additionally, the addition of other polymeric precursors was found to compromise the solubilization and increase the viscosity of the formulations, particularly in the case of MCCA. Therefore, to ensure comparability between samples, the concentration of GelMA was fixed at 10% (*w*/*v*), while all other polymers were maintained at 2.5% (*w*/*v*) for further tests.

### 3.3. Processing of the Formulations by DLP and Characterization of the 3D Constructs

**Figure 5:**
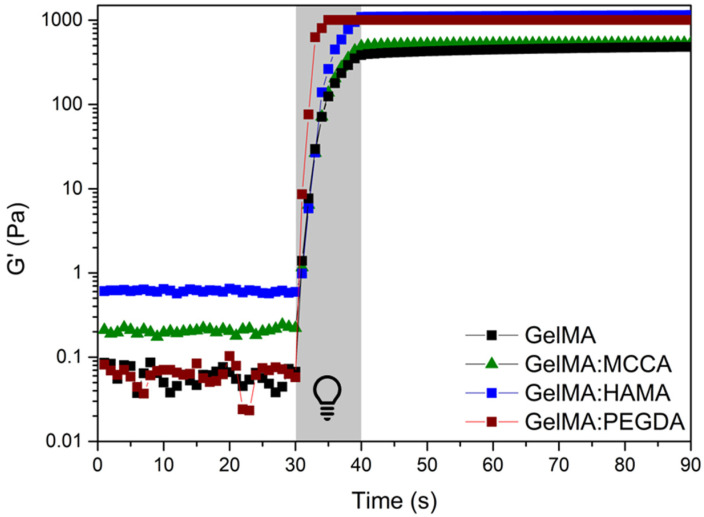
Time-sweep curves showing the evolution of the storage modulus (G’) of formulations used for DLP 3D printing. The UV light was turned on after a 30 s delay, and the G’ values were recorded over time.

To confirm the applicability of the formulations in the printing process, first, the formulations were subjected to photorheology tests to assess their behavior under UV light exposure, providing crucial insights into their photocrosslinking and viscosity properties. The time-sweep curves showing the evolution of the storage modulus (G’) of the tested formulations are presented in Figure 5.

The G’ of the resins can provide information about their behavior in two different stages: before exposure to light—the first 30 s of the graphic; and under exposure to light—the time interval of 30–40 s represented in the gray area. As seen in the first part of the graph, the GelMA and GelMA:PEGDA exhibited similar and lower viscosities, while GelMA:MCCA and in particular GelMA:HAMA presented a higher viscosity. Despite this increased viscosity, the formulations presented a rapid response to light irradiation, indicating suitable properties for application in DLP printing [40].

Additionally, as already observed in the preliminary curing tests, the addition of the HAMA and PEGDA precursors led to faster curing kinetics.

Next, the four chosen formulations were subjected to the 3D printing process by DLP, using the same printing parameters.

The printability of each formulation was evaluated by the fabrication of constructs with complex designs and different surface morphologies. The production of microneedle-like structures with features in the micron range usually offers better adhesion, and they have been reported to be useful for minimally painful wound management. Moreover, the protruding surface of these systems ensures direct contact with the wound and increases the surface contact area, improving the conditions for the healing process [41]. Additionally, this also provides an opportunity to evaluate the printing fidelity and resolution offered by each formulation.

Overall, all formulations proved to be able to produce 3D constructs with relatively high print fidelity in accordance with the intended designs. However, some differences were observed during the printing process and in the final printed products.

Among all formulations, the GelMA formulation presented the greatest challenge in terms of printability and in achieving structures with high resolution and definition. The main issue encountered was related to the thermoresponsive nature of the polymeric precursor. Due to the physical crosslinking of the GelMA precursors, during the immersion and lifting of the platform in the resins’ vat, air bubbles were formed and consequently trapped between the light source and the platform. This led to constructs with lower definition, as the air bubbles hindered proper curing of the resin in the intended place. This problem was effectively mitigated by incorporating other polymers in the formulation. The GelMA:MCCA formulations, in comparison to the others, exhibited a slightly opaque appearance and increased viscosity during printing. However, these characteristics did not significantly compromise the resolution of the final printed constructs. Both the GelMA:HAMA and GelMA:PEGDA formulations demonstrated excellent printability, enabling the fabrication of highly detailed microstructures with high resolution and precision, without major difficulties. This can be explained by the faster crosslinking kinetics observed in the photorheology tests.

**Figure 6:**
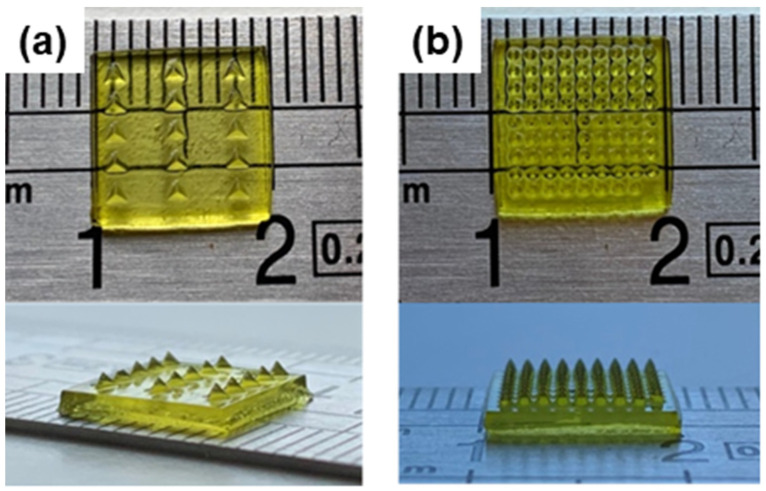
The 3D-printed GelMA:PEGDA patches: Micro-pyramids (**a**) and Micro-spikes (**b**).

Figure 6 shows the macroscopic images of the 3D-printed constructs obtained with the GelMA:PEGDA formulation.

#### 3.3.1. Surface Morphology

**Figure 7:**
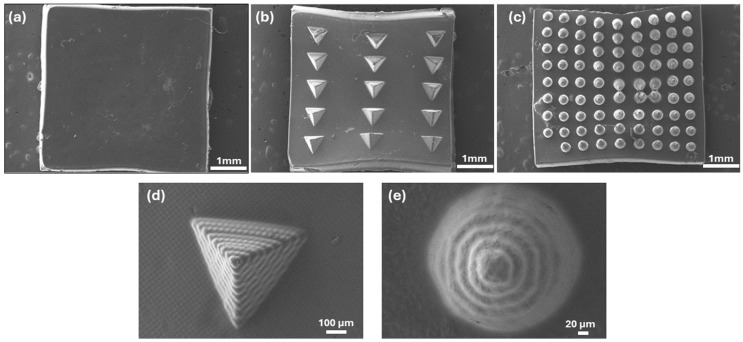
SEM images of the 3D-printed models using the GelMA:PEGDA formulation: (**a**) Smooth-surface patch; (**b**) Micro-pyramids patch; (**c**) Micro-spikes patch (magnification of 17 X, scale bar = 1 mm); (**d**) Micro-pyramid (magnification of 100 X, scale bar = 100 μm); and (**e**) Micro-spike (magnification of 250 X, scale bar = 20 μm).

The 3D-printed constructs were also analyzed via SEM (Figure 7). The SEM micrographs demonstrate the high printing quality and high resolution of the DLP printer with the prepared resin. The distinct whole-layer curing mechanism allows the printed constructs to have a smooth and uniform surface finish, as seen in Figure 7a. The printing quality of the microstructures was also evaluated with two different models: micro-pyramids and micro-spikes (Figure 7b,c, respectively). Both models displayed highly detailed structures with very precise layers of the desired geometry.

#### 3.3.2. Gel Content

The gel content of the 3D constructs was evaluated, and the results are presented in Table 4.

The gel content of the GelMA hydrogels was considerably lower than that of hydrogels with other polymers added in the formulation. This behavior is expected, since GelMA has a lower crosslinking capacity in comparison to the other polymers tested. Therefore, the incorporation of the HAMA and PEGDA precursors in the DLP formulations proved to increase the crosslinking efficiency of the printed hydrogel by having a gel content higher than 90%. This phenomenon can be attributed to the faster crosslinking kinetics of the formulations, likely resulting from the lower molecular weight of the two precursors, particularly PEGDA.

Meanwhile, the gel content of the GelMA:MCCA hydrogels was significantly lower than expected. This approximate loss of 20% of gel is due to the insufficient crosslinking process that occurs with the MCCA polymer. The lack of crosslinking in the GelMA:MCCA formulation can be explained by the less reactive nature of the allylic group.

#### 3.3.3. Hydrolytic Degradation

**Figure 8:**
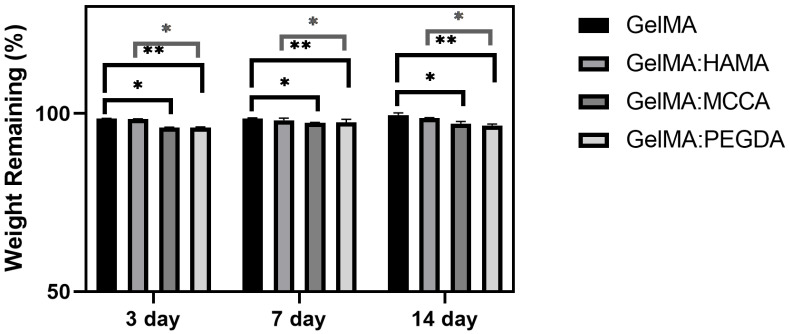
Weight remaining (%) of the printed constructs after immersion in PBS solution at 37 °C. (One-way analysis of variance (ANOVA) with Tukey’s multiple comparison test. Values of *p* < 0.05 are represented with *; values of *p* < 0.001 are represented with **).

For the envisaged application, it is expected that the printed patches can maintain their structural integrity and mechanical properties for at least two weeks [42]. The in vitro hydrolytic degradation behavior of the hydrogels is shown in Figure 8. All the tested patches presented a low value of weight loss in the tested medium. After a 14-day period of incubation, the maximum weight loss observed was 4%. At the end of the test, the hydrogels appeared to maintain the structural integrity, being suitable for the intended application.

### 3.4. Functionalization of the 3D Constructs with Gallic Acid (GA) to Provide Antioxidant Properties

Hydrogels with antioxidant properties play a crucial role in regulating the wound healing process. While low levels of reactive oxygen species (ROS) are necessary for proper wound healing, as they contribute to defense against external damage, excessive ROS can interfere with the normal healing process [43]. High levels of ROS trigger an excessive inflammatory response, which in turn leads to further ROS production. This vicious cycle creates a redox imbalance that is both characteristic of and responsible for non-healing wounds [44]. Therefore, incorporating antioxidant properties into hydrogels for wound dressing is important to maintain redox homeostasis and promote normal wound healing.

Recent studies have demonstrated that GA exhibits a wide range of pharmacological activities, including antimicrobial, anti-inflammatory, antioxidant, antitumor and antiaging effects [21].

The functionalization of the different constructs with GA was achieved by reacting the -COOH groups of GA with the -NH_2_ groups of GelMA, mediated by EDC/NHS activation chemistry (Scheme 4). It is reported that the incorporation of phenolic compounds such as GA into GelMA-based hydrogels significantly affects the properties of the functionalized hydrogels, namely the swelling capacity and their mechanical properties under compression; the swelling capacity typically suffers a reduction, while the values of compressive strength and compressive modulus increase [45,46]. These changes in properties are justified by the formation of additional hydrogen bonding between the hydrogel chains and the OH groups of the phenolic compounds. This increased hydrogen bonding strengthens the cohesion of the network, resulting in a reduction in pore size between the crosslinked polymer chains [24]. In the case of the GelMA-based constructs prepared in this work, a notorious decrease was noted in the size of the construct after the functionalization with GA (Figure S5), suggesting the formation of a more cohesive network.

#### Antioxidant Properties

**Figure 9:**
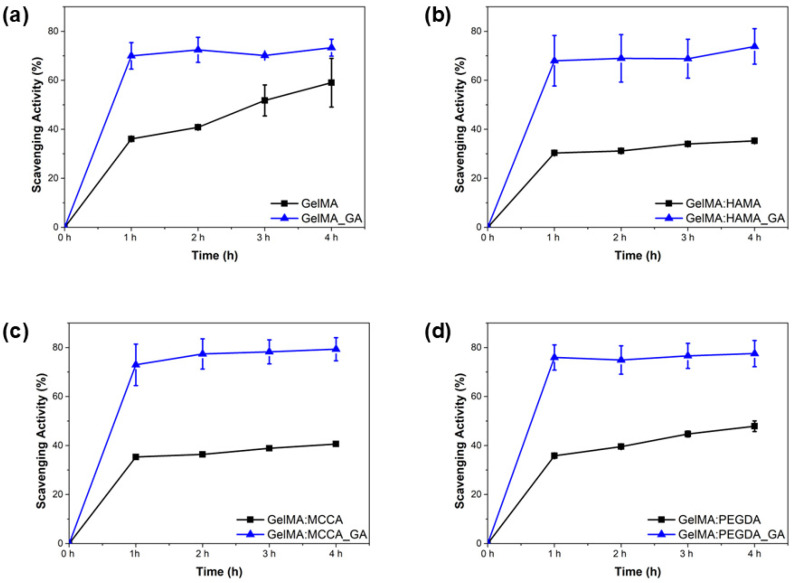
DPPH clearance spectra of GelMA (**a**), GelMA:HAMA (**b**), GelMA:MCCA (**c**) and GelMA:PEGDA (**d**).

The GA-functionalized printed constructs were evaluated in terms of their antioxidant capacity using the DPPH free radical scavenging assay. The reduction in DPPH free radicals was quantified via UV–VIS spectroscopy (Figure 9).

All printed constructs proved to have antioxidant capacity, achieving a free radical reduction close to 80%, after only 4 h of immersion. It is also possible to observe that most of the reduction occurred during the first hour of immersion, which suggests that the functionalization of the 3D constructs with GA endowed them with ROS scavenging properties.

This behavior was not completely unexpected, since GA has been shown to have strong antioxidant capabilities due to the hydrogen atom donation provided by the triple phenolic hydroxyl groups present in the GA structure [21,47]. However, the reduction was not expected to occur so rapidly. This result can be explained by the high concentration of GA used to functionalize the hydrogels. It can also be observed that the unmodified hydrogels showed some antioxidant properties. Since they all present similar reduction values, this may be provided by the common factor, GelMA. It is reported that the numerous hydroxyl groups in GelMA can offer some antioxidant properties [48].

### 3.5. Adhesion Capacity of the 3D Constructs

Adhesion is one of the key requirements and characteristics that a wound dressing must possess to ensure proper function and effectiveness [4,49]. However, the adhesive strength of a wound dressing must be carefully balanced. The dressing should not adhere too strongly to the wound site, as this can cause pain during removal. At the same time, it must exhibit sufficient adhesion to stay in place and provide effective protection and support throughout the healing process [7,50].

First, the adhesive capacity of the 3D-printed constructs was evaluated visually, making use of different substrates (metal, glass, paper, the outer layer of porcine skin (epidermis) and the inner layer of porcine skin (dermis)). This first assessment focused on evaluating the adhesion capacity and the influence of the different surface morphologies of the printed constructs. It is reported that the presence of mechanical interlockings can enhance the interaction between the hydrogel and the surface, improving its overall adhesion [46,51]. The incorporation of micro-punching structures on the hydrogel surface creates micro-protrusions in the tissue, which prevent easy separation of the hydrogel from the tissue. Additionally, this increases the contact surface area of the hydrogels, potentially enhancing adhesion. Literature reports suggest that the inclusion of micro-pyramids can improve the adhesive capacity of the hydrogel by up to 50% compared to hydrogels with a smooth and flat surface [49].

**Figure 10:**
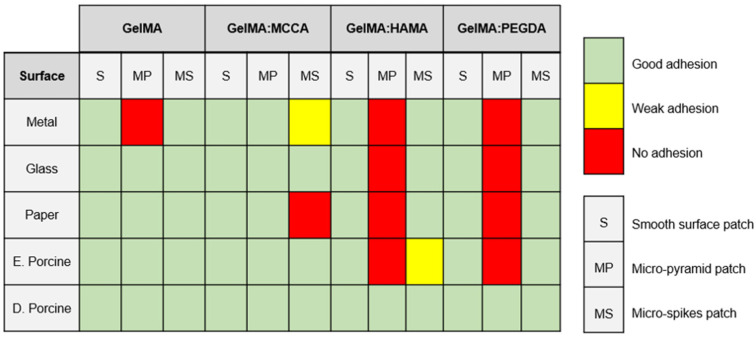
Adhesion capacity of the different surface models of each formulation on various contact surfaces.

The adhesive response of the different printed models of each formulation to these contact surfaces is presented in Figure 10.

It can be observed that, in general, all formulations exhibited high adhesive capacity on various contact surfaces and, importantly, adhered well to the skin. This can be attributed to the presence of several chemical groups on the hydrogels’ surface (e.g., -OH, -NH_2_, -C=O) that can form hydrogen bonds with the functional groups present in the tissue, enhancing hydrogel adhesion to the skin [52].

In this study, the incorporation of the two microstructures yielded different outcomes. The micro-pyramid structure resulted in reduced adhesion, particularly for the GelMA:HAMA and GelMA:PEGDA formulations, while the micro-spikes showed no significant difference compared to the smooth surface. This can be mainly explained by the differences in design of the two microstructures. Instead of increasing the contact area, the distance between peaks in the micro-pyramid design resulted in the opposite effect.

The adhesiveness of the printed hydrogels is influenced by the presence or incorporation of functional groups in the polymer structure. When these functional groups come into contact with tissue, they interact and bind, leading to adhesion. This interaction can occur through two main mechanisms: physical forces, such as hydrogen bonding or van der Waals forces, or chemical forces, which involve covalent bonding between the hydrogel and the tissue [46,51,53].

The functionalization of the patches with GA will incorporate new phenolic groups into the polymer structure, providing more binding sites with the contact surface, which is expected to enhance the adhesiveness of the hydrogels [54]. This increase in adhesive capacity was observed during the experiment, particularly in the GelMA:HAMA and GelMA:PEGDA hydrogels. The hydrogels that initially showed no adhesion to the patterned micro-pyramid models on most contact surfaces successfully adhered to all contact surfaces when functionalized with GA. This confirmed that modifying the hydrogels with GA enhanced their adhesion capacity.

The application of an iron metal ion (Fe^3+^) solution was also tested and resulted in an improvement in the adhesive capacity of the hydrogels. This enhancement was particularly noticeable in the GA-modified hydrogels. The galloyl groups of GA incorporated into the hydrogel structure are derivatives of catechol groups, which have been shown to enhance the adhesion capacity of hydrogels through coordination with Fe^3+^ ions [49]. However, the adhesiveness of the unmodified hydrogels also improved with the application of the Fe^3+^ solution in the absence of GA (Figure S6). This can be attributed to the interaction between the Fe^3+^ ions and the hydroxyl, carboxyl and amine groups present in the polymers composing the patches [54].

All these results show that adhesion can be improved via functionalization of the patches with GA, followed by the addition of Fe^3+^. This treatment not only allowed the patches to adhere effectively to porcine skin but also enabled them to withstand movement and folding of the skin, as seen in Figure S7 and Video S1.

The adhesiveness of the two best-performing formulations (GelMA and GelMA:MCCA) was evaluated using a pull-away test. In this test, tackiness and adhesion were assessed by measuring the normal force over time as the upper plate was retracted from the hydrogel patches. This test was conducted to compare the adhesion properties of non-functionalized hydrogels with those functionalized with GA and by the combination of GA and Fe^3+^ ions. The adhesive strength behavior of the patches was determined, and the values are reported in Table 5.

All tested samples exhibit good adhesivity, which can be significantly enhanced by the incorporation of GA. This functionalization increases adhesivity strength by more than 100%, which can be further enhanced through the addition of Fe^3+^ ions. Moreover, the results suggest that the presence of MCCA may slightly enhance adhesive performance, possibly due to the formation of additional physical crosslinks, such as hydrogen bonds [55].

The adhesiveness of the developed systems can be attributed to non-covalent interactions between the hydrogel constructs and the surface, including metal coordination and electrostatic interactions. Additionally, the phenolic groups from GA can further enhance these interactions by providing additional binding sites, contributing to an improved adhesive performance [56].

### 3.6. In Vitro Cytotoxicity

**Figure 11:**
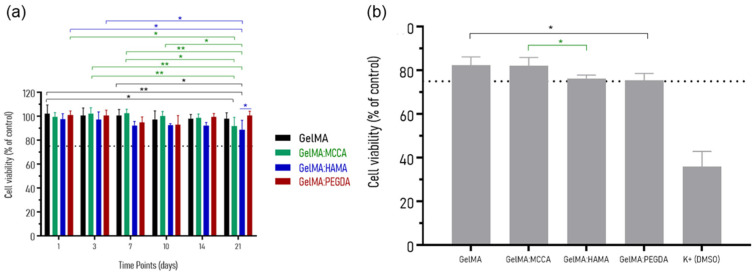
In vitro cytocompatibility assay: (**a**) Indirect cytotoxicity assay showing cell viability (% relative to untreated cells) at different time points using 100% extract; (**b**) Direct cytotoxicity assay showing the number of viable NHDF cells cultured onto hydrogel surface at day 3. Each column represents the results of the experiments repeated three times. (One-way analysis of variance (ANOVA) with Tukey’s post hoc test. Values of *p* < 0.05 (*) and *p* < 0.001 (**) were considered significant.)

For the indirect test, NHDF cells were cultured in contact with the extracts of the degradation products of the printed hydrogel formulations after incubation times of 1, 3, 7, 10, 14 and 21 days. The result of the indirect test is shown in Figure 11a as a percentage of cell viability (%). The untreated cells cultured on the surface of the wells (without extracts of the degradation products) served as a control. According to ISO 10993-5:2009, materials with cell viability greater than 75% can be considered non-cytotoxic [32]. As can be seen, the cell viability of NHDF cells cultured in contact with the extracts was always greater than 75% at the different time points for all formulations, which indicates that the degradation products are non-cytotoxic. The extracts presented a cell viability percentage between 88 and 102% from day 1 to day 21, with the highest viability for GelMA (98.06–102.20%), followed by GelMA:PEGDA (93.04–101.10%), GelMA:MCCA (91.87–102.41%) and GelMA:HAMA (88.55–97.49%). Therefore, these data indicate that the processing of the printed structures does not lead to the leaching of soluble cytotoxic compounds, proving their safety and applicability. Additionally (direct contact), NHDF cells were seeded on the surface of the printed hydrogels and on polystyrene cell culture plastic as a control. After 3 days of incubation (Figure 11b), the cell viability for cultures with GelMA and GelMA:MCCA hydrogels was approximately 82% compared to untreated cells. However, the mean cell viability values were significantly lower for the GelMA:HAMA (76.41%) and GelMA:PEGDA (75.53%) hydrogels, and a significant statistical difference was noted between GelMA and GelMA:PEGDA and between GelMA:MCCA and GelMA:HAMA (*p* < 0.05). This can possibly be due to the high crosslinking density and stiffness of GelMA:HAMA and GelMA:PEGDA constructs, which can limit cell adhesion, proliferation and nutrient diffusion.

## 4. Conclusions

This study developed and characterized 3D-printed hydrogel constructs designed for personalized wound dressing applications. Using the high-resolution capabilities of DLP printing, hydrogel patches with different surface microstructures and compositions were produced. To improve the properties of GelMA-based hydrogels, various polymers were functionalized and combined to prepare the DLP formulations. The resulting formulations, comprising GelMA, HAMA, PEGDA and MCCA, presented a rapid crosslinking and high crosslinking efficiency, allowing the fabrication of high-resolution micropatterned structures. Additionally, the functionalization with GA significantly enhanced the antioxidant properties of the printed patches, achieving over 70% DPPH clearance, while adhesion studies underscored the superior binding capability of GelMA and GelMA:MCCA, particularly in micro-spike surface models. Moreover, the introduction of GA in combination with Fe^3+^ ions further improved adhesiveness of the materials. In vitro cytocompatibility assays showed that the 3D-printed constructs did not elicit adverse effects on the tested cell line.

In conclusion, this study confirms the potential of using natural polymer-based formulations for DLP to create 3D-printed constructs for advanced wound healing applications. These hydrogels may open new perspectives in the field, particularly when combined with therapeutic agents to enhance recovery. Additionally, exploring alternative structures and morphologies could further optimize their performance. However, further studies are required to assess their in vivo performance and long-term biocompatibility to confirm their clinical relevance and identify the most effective formulations.

## Figures and Tables

**Table 1:** Concentration of polymers, photoinitiator and photoabsorber in DLP formulations.

DLP Formulation	Precursor (wt.%)	PI (wt.%)	PA (wt.%)
GelMA	12.5% GelMA	0.50	0.05
GelMA:MCCA	10% GelMA + 2.5% MCCA		
GelMA:HAMA	10% GelMA + 2.5% HAMA		
GelMA:PEGDA	10% GelMA + 2.5% PEGDA		

**Table 2:** Degree of functionalization of the functionalized polymeric precursors.

Hydrogel	DS (%)
GelMA	51.5
HAMA	37.3
MCCA	27.1

**Table 3:** Curing time of the polymeric precursors at different polymer concentrations.

Polymeric Precursor	Polymer Concentration % (*w*/*v*)	Curing Time (s)	Obs.
GelMA	2.5	No gelation	-
	5	120	Weak and sticky hydrogel
	10	60	Soft and sticky hydrogel
	15	60	Stiff and sticky hydrogel
	20	60	Stiff hydrogel
HAMA	2.5	120	Soft hydrogel
	5	120	Soft hydrogel
	10	120	Elastic hydrogel
	15	30	Stiff hydrogel
	20	30	Stiff hydrogel
MCCA	2.5	120	Elastic hydrogel
	5	120	Elastic hydrogel
	10	120	Stiff hydrogel
	15	-	No dissolution of the precursor
	20	-	No dissolution of the precursor
PEGDA	2.5	No gelation	-
	5	120	Soft and elastic hydrogel
	10	60	Elastic hydrogel
	15	60	Stiff hydrogel
	20	30	Stiff hydrogel

**Table 4:** Gel content of the hydrogels (average of five valid tests).

Hydrogel	Gel Content (%)
GelMA	87.51 ± 1.50
GelMA:MCCA	81.22 ± 0.84
GelMA:HAMA	92.30 ± 0.40
GelMA:PEGDA	94.89 ± 0.30

**Table 5:** Comparison of the adhesivity and normal force peak of the GelMA and GelMA:MCCA hydrogels without functionalization, those functionalized with GA and by the combination of GA and Fe^3+^ ions.

Hydrogel	Adhesiveness (N.s)
GelMA	2.02 ± 0.86
GelMA + GA	4.53 ± 0.46
GelMA + GA + Fe	5.21 ± 0.47
GelMA:MCCA	2.68 ± 0.07
GelMA:MCCA + GA	4.69 ± 0.14
GelMA:MCCA + GA + Fe	5.57 ± 0.10

## Data Availability

The original contributions presented in this study are included in the article/Supplementary Material. Further inquiries can be directed to the corresponding authors.

## Supplementary Materials

The following supporting information can be downloaded at: https://www.mdpi.com/article/10.3390/polym17081114/s1, Figure S1: CAD model of the developed patches with dimensions. (a) micro-pyramid and (b) micro-spikes; Figure S2: ^1^H NMR spectra of GelMA and Gelatin, in D_2_O. The degree of sustitution (DS) was according was calculated according to the equation; Figure S3: ^1^H NMR spectrum of HAMA, in D_2_O. The DS was according was calculated according to the equation; Figure S4: ^1^H NMR spectrum of MCCA, in D_2_O. The DS was calculated according to the equation; Figure S5: Visual comparison of hydrogel size reduction, showing the non-functionalized hydrogel (a) and the GA-functionalized hydrogel (b); Figure S6: Non-functionalized hydrogel (a), non-functionalized hydrogel with Fe^3+^ solution (b) and GA functionalized hydrogel Fe^3+^ solution (c); Figure S7: Adhesive properties of microstructured 3D-printed hydrogels evaluated on porcine skin. (a) functionalised with GA and (b) functionalised with GA and Fe^3+^ ions; Video S1: Adhesive properties of the hydrogel patches to porcine skin.
