# Supplementary material for: Light-Mediated 3D-Printed Wound Dressings Based on Natural Polymers with Improved Adhesion and Antioxidant Properties
Source: Polymers (Basel). 2025 Apr 20;17(8):1114. doi: 10.3390/polym17081114 (PMC12031436; doi:10.3390/polym17081114)
Supplement: Supplementary file 1 [file polymers-17-01114-s001.zip › polymers-3588011-supplementary/polymers-3588011-supplementary SI_CTP_v2.pdf]

Supporting Information

Light-mediated 3D-printed wound dressings based on natural polymers with improved adhesion and antioxidant properties

Rute Silva,[a] Matilde Medeiros,[a] Carlos T.B. Paula,[a,b*] Sofia Saraiva,[a] Rafael C. Rebelo,[a] Patrícia Pereira,[a,b] Jorge F.J. Coelho,[a,b] Arménio C. Serra,[a] Ana C. Fonseca[a*]

[a]*University of Coimbra, CEMMPRE, ARISE, Department of Chemical Engineering, Rua Sílvio Lima-Polo II, 3030-790 Coimbra, Portugal*

[b]*IPN, Instituto Pedro Nunes, Associação para a Inovação e Desenvolvimento em Ciência e Tecnologia, Rua Pedro Nunes, 3030-199 Coimbra, Portugal*

*Corresponding author:

Carlos T.B. Paula: cpaula@ipn.pt

Ana C. Fonseca: anafs@eq.uc.pt

Authors' e-mails:

Rute Silva: r rute.silva@student.uc.pt

Matilde Medeiros: medeirosmatilde07@gmail.com

Sofia Saraiva: uc44571@uc.pt

Rafael C. Rebelo: rafaelr@eq.uc.pt

Patrícia Pereira: papereira@ipn.pt

Jorge F. J. Coelho: jcoelho@eq.uc.pt

Arménio C. Serra: aserra@eq.uc.pt

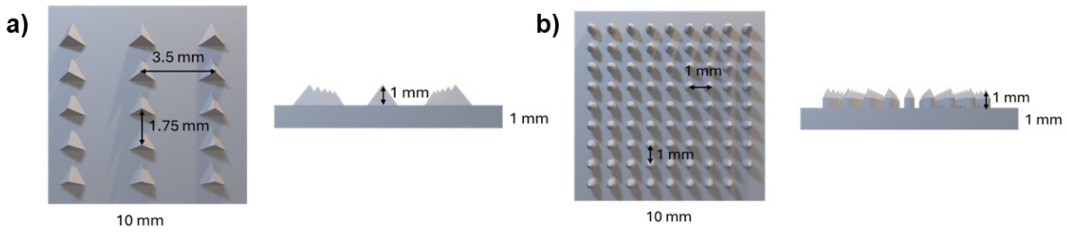

Figure S1. CAD model of the developed patches with dimensions. a) micro-pyramid and b) micro-spikes.

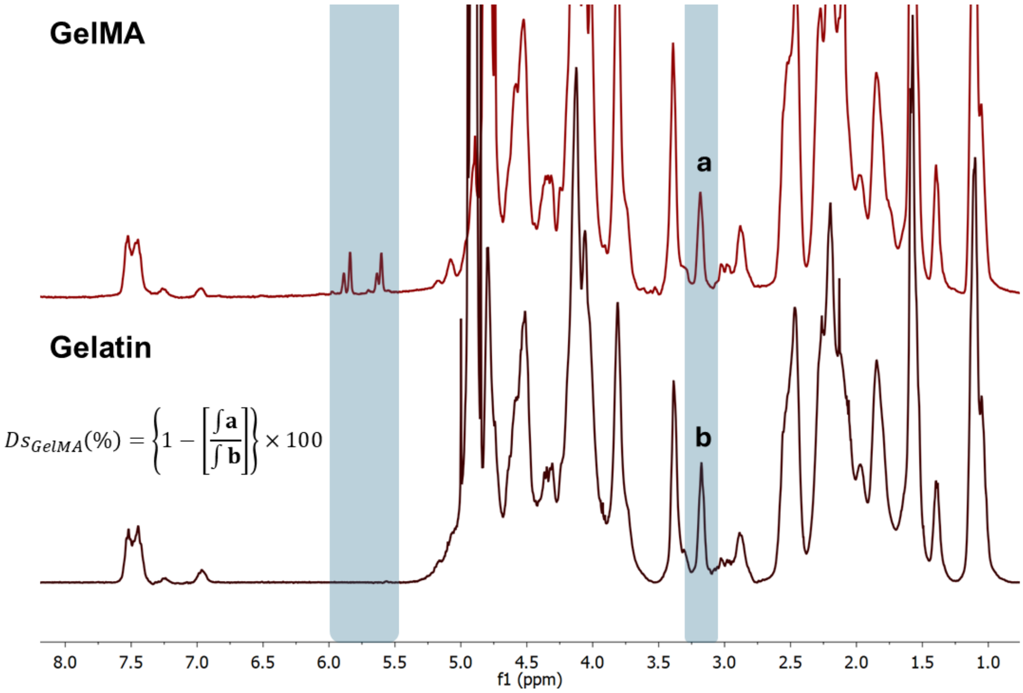

Figure S2. ^{1}H NMR spectra of GelMA and Gelatin, in D_2O. The degree of sustitution (DS) was according was calculated according to the equation.

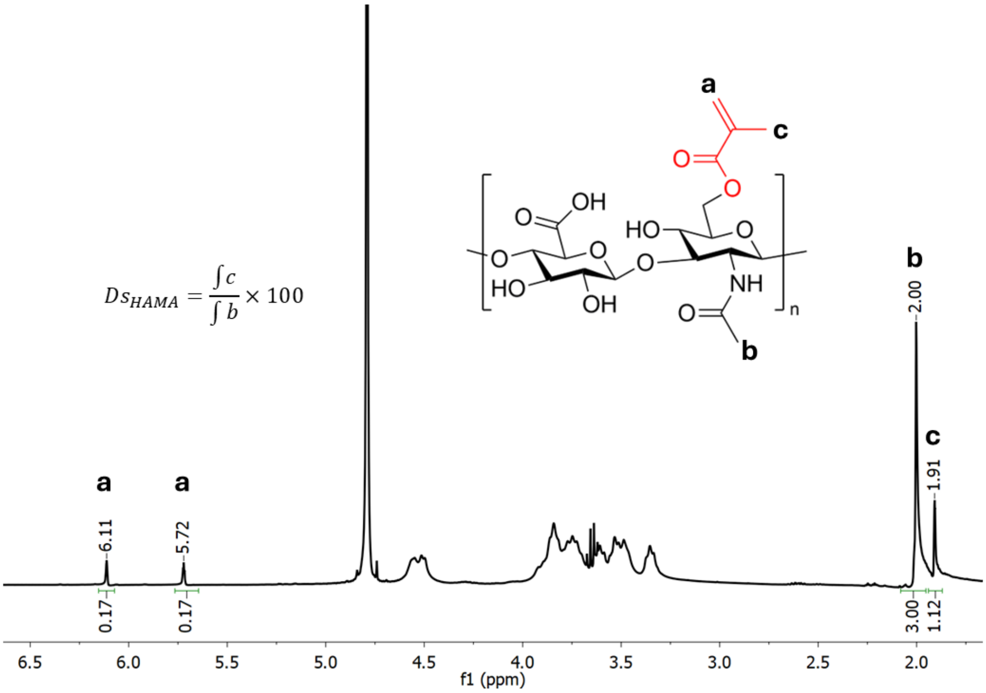

Figure S3. ^{1}H NMR spectrum of HAMA, in D_2O. The DS was according was calculated according to the equation.

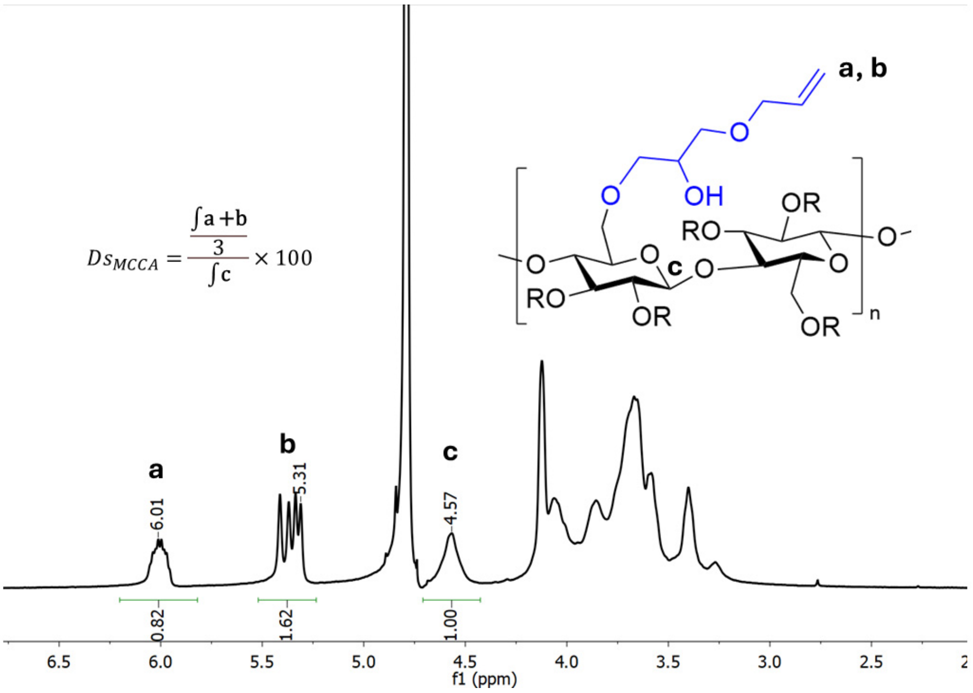

Figure S4. ^{1}H NMR spectrum of MCCA, in D_2O. The DS was calculated according to the equation.

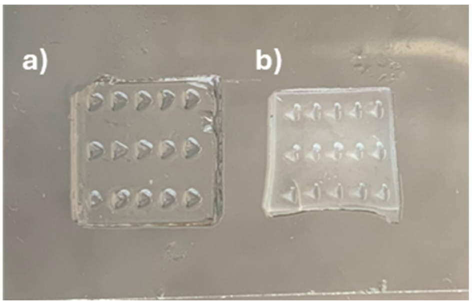

Figure S5. Visual comparison of hydrogel size reduction, showing the non-functionalized hydrogel (a) and the GA-functionalized hydrogel (b).

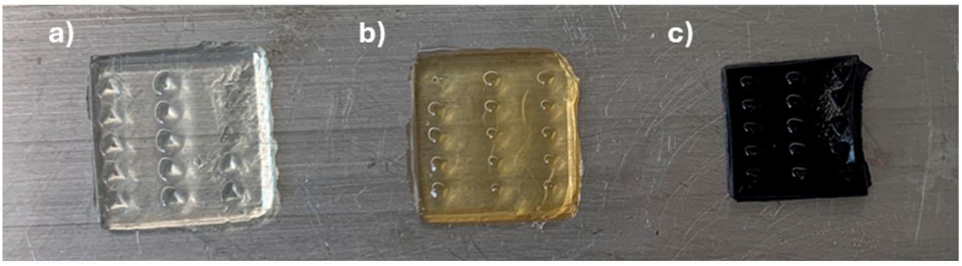

Figure S6. Non-functionalized hydrogel (a), non-functionalized hydrogel with Fe^{3+} solution (b) and GA functionalized hydrogel Fe^{3+} solution (c).

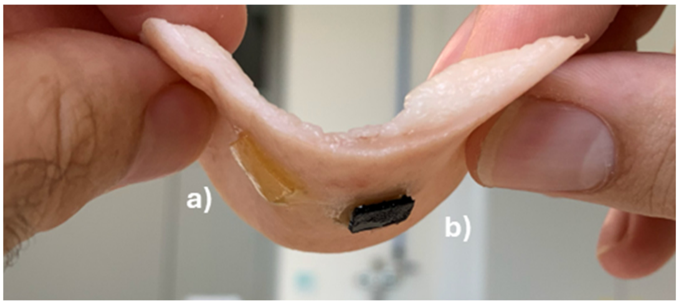

Figure S7. Adhesive properties of microstructured 3D-printed hydrogels evaluated on porcine skin. a) functionalised with GA and b) functionalised with GA and Fe^{3+} ions.
